# Supplementary material for: Modelling Skylarks (Alauda arvensis) to Predict Impacts of Changes in Land Management and Policy: Development and Testing of an Agent-Based Model
Source: PLoS One. 2013 Jun 6;8(6):e65803. doi: 10.1371/journal.pone.0065803 (PMC3675089; doi:10.1371/journal.pone.0065803)
Supplement: Supporting Information S4 — The skylark ODdox as a zipped archive. (ZIP) [file pone.0065803.s004.zip › Skylark_ODdox/class_sk_qual_grid.html]

ALMaSS Skylark ODdox: SkQualGrid Class Reference


ALMaSS Skylark ODdox
 2.0


- Main Page
- Related Pages
- Classes
- Files

- Class List
- Class Index
- Class Hierarchy
- Class Members

Public Member Functions |
Public Attributes

SkQualGrid Class Reference

`#include <skylarks_all.h>`

List of all members.

|  |  |
| --- | --- |
| Public Member Functions | |
| int | Insert (int a\_poly) |

|  |  |
| --- | --- |
| Public Attributes | |
| vector< int > | m\_polys |
| double | m\_qual |
| vector< int > | m\_sizes |

---

## Member Function Documentation

|  |  |  |  |  |  |
| --- | --- | --- | --- | --- | --- |
| int SkQualGrid::Insert | ( | int | *a\_poly* | ) |  |

inline

Referenced by SkTerritories::PreEvaluateQualGrid().

{

int l\_size = (int) m\_sizes.size();

m\_sizes.resize( l\_size + 1 );

m\_polys.resize( l\_size + 1 );

m\_polys[l\_size] = a\_poly;

m\_sizes[l\_size] = 1;

return l\_size;

}

---

## Member Data Documentation

vector< int > SkQualGrid::m\_polys

Referenced by SkTerritories::PreEvaluateQualGrid(), SkTerritories::PreFillQualCache(), and SkTerritories::PreFillTerrPolyLists().

double SkQualGrid::m\_qual

Referenced by SkTerritories::PreEvaluateHabitat(), SkTerritories::PreEvaluateHabitatStripX(), SkTerritories::PreEvaluateHabitatStripY(), and SkTerritories::PreEvaluateQualGrid().

vector< int > SkQualGrid::m\_sizes

Referenced by SkTerritories::PreEvaluateQualGrid(), and SkTerritories::PreFillTerrPolyLists().

---

The documentation for this class was generated from the following file:

- skylarks\_all.h


- SkQualGrid
- Generated on Thu Jan 10 2013 13:15:37 for ALMaSS Skylark ODdox by
   1.8.1.1
